# Aspects of Dynamic Balance Responses: Inter- and Intra-Day Reliability

**DOI:** 10.1371/journal.pone.0136551

**Published:** 2015-09-04

**Authors:** Daniel Schmidt, Andresa M. C. Germano, Thomas L. Milani

**Affiliations:** Department of Human Locomotion, Institute of Human Movement Science & Health, Technische Universitaet Chemnitz, Chemnitz, Saxony, Germany; West Virginia University, UNITED STATES

## Abstract

The Posturomed device is used as a scientific tool to quantify human dynamic balance ability due to unexpected perturbations, and as a training device. Consequently, the question arises whether such measurements are compromised by learning effects. Therefore, this study aimed to analyze inter- and intra-day reliability of dynamic balance responses using the Posturomed. Thirty healthy young subjects participated (24.3±3.2 years). The Posturomed was equipped with a triggering mechanism to enable unexpected, horizontal platform perturbations. A force platform was used to quantify Center of Pressure (COP) excursions for two time intervals: interval 1 (0–70 ms post perturbation) and interval 2 (71–260 ms post perturbation). Dynamic balance tests were performed in single leg stances in medio-lateral and anterior-posterior perturbation directions. Inter- and intra-day reliability were assessed descriptively using Bland-Altman plots and inferentially using tests for systematic error and intra-class-correlations. With regard to the mean COP excursions for every subject and all intervals, some cases revealed significant differences between measurement sessions, however, none were considered relevant. Furthermore, intra class correlation coefficients reflected high magnitudes, which leads to the assumption of good relative reliability. However, analyzing inter- and intra-day reliability using Bland-Altman plots revealed one exception: intra-day comparisons for the anterior-posterior direction in interval 2, which points towards possible learning effects. In summary, results reflected good overall reliability with the exception of certain intra-day comparisons in the anterior-posterior perturbation direction, which could indicate learning effects in those particular conditions.

## Introduction

The ability to successfully balance the human body is required in almost all everyday situations, and is important to avoid falls and restrictions of daily activities [1]. Mechanisms of human balance regulation are highly demanding, including three cooperating systems: the visual, vestibular and somatosensory system [2]. Interactions between these three systems vary considerably when comparing quasi-static to dynamic balance conditions [3]. Dynamic balance tests might be more useful to detect possible balance strategies [4], whereas quasi-static balance tests do not seem to be the optimal indicator for functional postural control [5]. Regarding dynamic balance, postural adjustments may be divided into anticipatory and compensatory responses [6]. Anticipatory responses are associated with strategies to preserve postural balance, hence preparing the body for a forthcoming perturbation [7]. Compensatory responses appear as direct muscular reactions responding to sensory feedback signals which are evoked by a perturbation that has already occurred.

Compensatory balance response patterns are present when on a train or a bus, which are induced by sudden accelerations or decelerations. Another example is when walking over ice-covered or wet, slippery surfaces. In all these examples, unexpected translational perturbations occur which are realistic balance challenges of daily life. These examples were taken as the basis of examining postural control strategies [8].

An extensive variety of such perturbation setups are implemented in experimental designs to cause a temporary disequilibrium. These are, for example, horizontal platform movements or video-linked force platforms [9–11]. Some of these devices, e.g. the Wii Balance Board (Nintendo, Kyoto, Japan), were shown to be both useful to quantify dynamic balance and reliable [11]. Another tool to induce standardized translational platform perturbations is the widely used Posturomed (Haider Bioswing GmbH, Germany) [12], which is also known to quantify dynamic postural control [12–17]. In this context, Taube et al. [5] used the Posturomed to assess dynamic balance ability after four weeks of slackline training using perturbed and unperturbed conditions. On the other hand, this device is also implemented for balance training and balance tasks [18], [19]. When training on the Posturomed, subjects or patients stand on one or two legs, or they perform different simultaneous tasks, like moving limbs etc. In one study, subjects stood on the Posturomed on their right leg for 40 s to evaluate postural stability [20]. In another study this device was used as a postural stabilization task, after subjects were asked to perform series of 3 trials of unexpected perturbations, with measuring intervals of 10 s [21]. Mierau et al. [18] used the Posturomed as a balance task device to analyze cortical activity in healthy subjects. They recorded three consecutive trials, each lasting 20 seconds with a resting period of one minute between trials. Kramer et al. [19] used the Posturomed as a training device in patients with multiple sclerosis. Training sessions lasted three weeks with a total of 9 training sessions, each lasting 30 minutes. Patients a) simply stood on the moveable platform or b) performed tasks with increasing difficulty on the Posturomed, e.g. standing on both legs, on toes or heels, on one leg or with external perturbations.

Due to the application of the Posturomed as a training tool, improvements of dynamic balance can be expected. Therefore, studies using this device to quantify dynamic balance ability might be prone to provoking balance improvements during data collection. In other words, potential learning effects may occur.

Despite these considerations, reliability aspects of the Posturomed have still not been extensively explored, although there is demand for reliable balance assessment tools [11]. To the knowledge of the authors, our study is the first which aimed to analyze the reliability of first dynamic balance responses after unexpected translational perturbations. Another study which dealt with a reliability analysis of the Posturomed was performed by Boeer et al. [22], but they did not induce unexpected perturbations. They concluded that the Posturomed exhibits slight learning effects, but still shows reproducible results to quantify balance ability. Due to its widespread range of application, a better understanding of reliability aspects is of fundamental importance. For this reason, the objective of the present study was to investigate the intra- and inter-day reliability of dynamic balance responses after unexpected perturbations using the Posturomed device, whereas low intra- and inter-day reliability was hypothesized.

## Materials and Methods

### Subjects

Thirty healthy, young subjects (15 females, 15 males) participated in this study (mean±SD: 24.3±3.2 yrs, 71.4±12.5 kg, 173.8±9.1 cm). Participants with a history of lower extremity pain or lower leg injury for at least six months before the measurements were excluded from this study. None of the subjects had any peripheral neuropathy or other similar disorders. Subjects gave their written informed consent. In case of any discomfort, participants were instructed to stop measurements. All procedures were executed in accordance with the recommendations of the Declaration of Helsinki. This study was approved by the Ethics Committee of the Faculty of Behavioural and Social Sciences of the corresponding university.

### Instrumentation and Testing Procedure

The Posturomed consists of a horizontally moveable bottom-platform which is vertically suspended. More recent versions of this device are equipped with a lever-based provocation unit to enable unexpected horizontal perturbations. Since the version of the Posturomed used in this study did not provide such a provocation unit, it was equipped with an electro-magnet which fixed the bottom-platform after shifting it 20 mm out of its neutral position. This kind of perturbation unit was also used in other previous studies [5], [21], [23]. Unexpected perturbations were induced by manually triggering the electro-magnet causing the bottom platform to swing until it reached the neutral position again. A force-platform (IMM Holding GmbH, Germany; 1 kHz) was installed directly on top of the bottom-platform. Furthermore, a single axis accelerometer ADXL78 (Analog Devices Inc., USA) was integrated into the setup to calculate the reversal points of the platform. Room temperature was controlled in accordance with EN ISO/IEC 17025 (23±2°C) and was monitored using a digital C28 type K thermocouple (Comark Instruments, U.K.). To guarantee foot temperature variations of less than ±5 to 6°C, which influence plantar sensibility and consequently movement coordination [24], a mini-flash infrared thermometer (TFA Dostmann GmbH & Co KG, Germany) was used to measure the temperature of the dominant foot sole before and after trials. Dynamic balance tests were performed in single leg stance (dominant leg) for two conditions: medio-lateral (ML) and anterior-posterior (AP) perturbation direction. For ML, subjects stood on top of the setup in such a way that the lateral aspect of the dominant foot was pointed towards the electro-magnet, causing an ML perturbation after its release. For AP, subjects were instructed to turn 90° so that the heel was pointed towards the electro magnet, causing an AP perturbation. The exact foot position on top of the setup was marked with tape to ensure higher standardization. For each condition (ML, AP), 12 trials were collected in a randomized order (randomization routine programmed in R, The R Foundation for Statistical Computing, Austria), resulting in a total of 24 trials for one complete measurement session. For data analysis, both conditions were then separated and brought into temporal order (AP 1, …, 12; ML 1, …, 12). In order to become accustomed to the apparatus, each subject performed six trials (three in each condition) before starting data collection. During the measurements, participants were asked to look straight ahead with their arms hanging loosely down at their sides. To analyze intra-day reliability, the entire testing procedure mentioned above was performed twice a day for each subject, in the morning and afternoon, with a break period of at least four hours, resulting in two data collections per day (1_1 and 1_2). The same procedure was repeated another day (2_1 and 2_2) with 48 hours off between day one and day two. Consequently, each subject took part in four measurement sessions (4x24 trials).

### Data Processing and Statistics

Data processing was conducted using R (The R Foundation for Statistical Computing, Austria) and center of pressure (COP) total excursions were calculated for two time intervals: 0–70 ms post trigger (Int 1) and 71–260 ms post trigger (Int 2). These corresponding first and second reversal points (70 and 260 ms, respectively) of the oscillating bottom-platform were calculated over all subjects resulting in (mean±SD) 70.3±4.6 ms (reversal point 1) and 259.8±15.3 ms (reversal point 2).

Descriptively data are presented as graphs and tables including individual data and means of the 12 trials and standard deviations. Additionally, Bland-Altman plots are depicted to assess absolute reliability. Since measurement error can be of systematic nature (e.g. bias) [25], a repeated measures Analysis of Variance (ANOVA) was performed with Bonferroni post hoc tests to detect significant bias [25], [26]. The level of significance was corrected due to the number of measurement sessions (n = 4) to α = 0.05/4 = 0.0125. The relevance of mean differences was determined by root mean square error (RMSE) calculations. To assess relative reliability, intra-class-correlation (ICC) coefficients were included. As this paper deals with test-retest reliability and averaged COP values, ICC model 3,k was used, as recommended [27]. Furthermore, to quantify data variability, coefficients of variation (COVs) were calculated.

## Results

**Fig 1:**
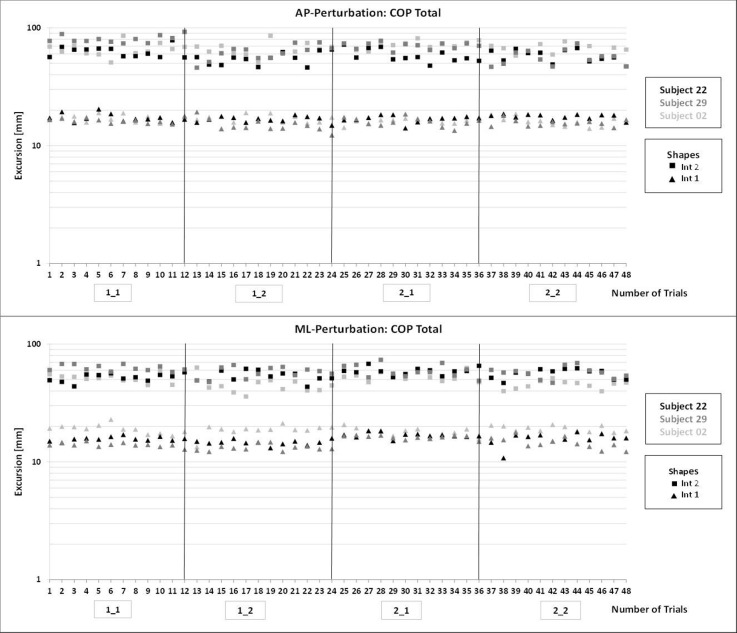
COP Total excursions in both perturbation directions: AP (top), ML (bottom) from six randomly chosen subjects, showing all individual trials (12) for each of the four measurement sessions (1_1, …, 2_2).

All trials (4 measurement sessions x 12 trials, respectively) were taken into consideration in both perturbation directions (AP, ML) and intervals; Fig 1.

Interval 1 exhibited ranges of COP Total excursions from 10–20 mm (96% of all subject trials) for both perturbation directions. Over the course of all 48 trials, no tendency of increasing or decreasing COP Total excursions was observed, see Fig 1.

For interval 2, measurements ranged from 40–90 mm (88% of all subject trials) for both perturbation directions (Fig 1). Considering individual measurement sessions and their 12 trials, for each condition, no tendency towards increasing or decreasing excursions was found for AP or ML. When comparing intra-day data (day one or day two), decreased COP excursions were evident for seven out of 30 subjects at the retests for AP. In ML, this finding was evident for three out of 30 subjects (e.g. see Fig 1, subject 02: 1_1 vs. 1_2, interval 2) and was not observed for inter-day comparisons.

**Fig 2:**
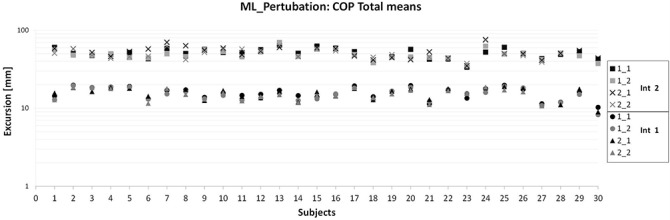
Mean COP Total excursions in the ML direction for all subjects, intervals and measurement sessions (1_1, …, 2_2).

Fig 2 shows descriptively that inter-subject variations occurred and were greater for interval 2, but also that no trend towards increasing or decreasing intra-subject excursions was present during measurement sessions, especially for interval 2 (e.g. mean excursions from 1_1 were not always greater than for 2_2).

Table 1 shows no significant differences for interval 1 in the AP direction. However, in the ML direction, significantly higher COP excursions were found for 1_1 compared to 1_2 and 2_2. In interval 2, no significant differences were present in the ML direction. For AP, comparisons with significantly decreased COP excursions at the retest were evident when comparing 1_1 vs. 1_2, 2_1 vs. 2_2 and 1_1 vs. 2_2.

**Table 1:** COP Total excursions (mean±SD) for both perturbation directions (AP, ML), all four measurement sessions and all analyzed intervals. Significant differences between the four measurement sessions are marked with superscripted symbols; see below (α = 0.0125).

AP COP Total [mm]	1_1	1_2	2_1	2_2
**Int 1**	15.1±1.9	14.8±1.8	15.1±1.8	14.7±2.2
**Int 2**	61.4±13.3* ^#^	54.2±12.2*	56.5±10.8^Φ^	52.8±10.6^#^ ^Φ^
**ML COP Total [mm]**				
**Int 1**	15.8±2.7 ^Ψ^ ^Χ^	15.1±2.8 ^Ψ^	15.6±2.7	15.1±2.7 ^Χ^
**Int 2**	51.1±7.3	49.0±7.3	52.4±8.9	50.4±8.0

Significant differences

*p˂0.001

^#^p = 0.001

^Φ^p = 0.003

^Ψ^p = 0.003

^Χ^p = 0.002.

**Fig 3:**
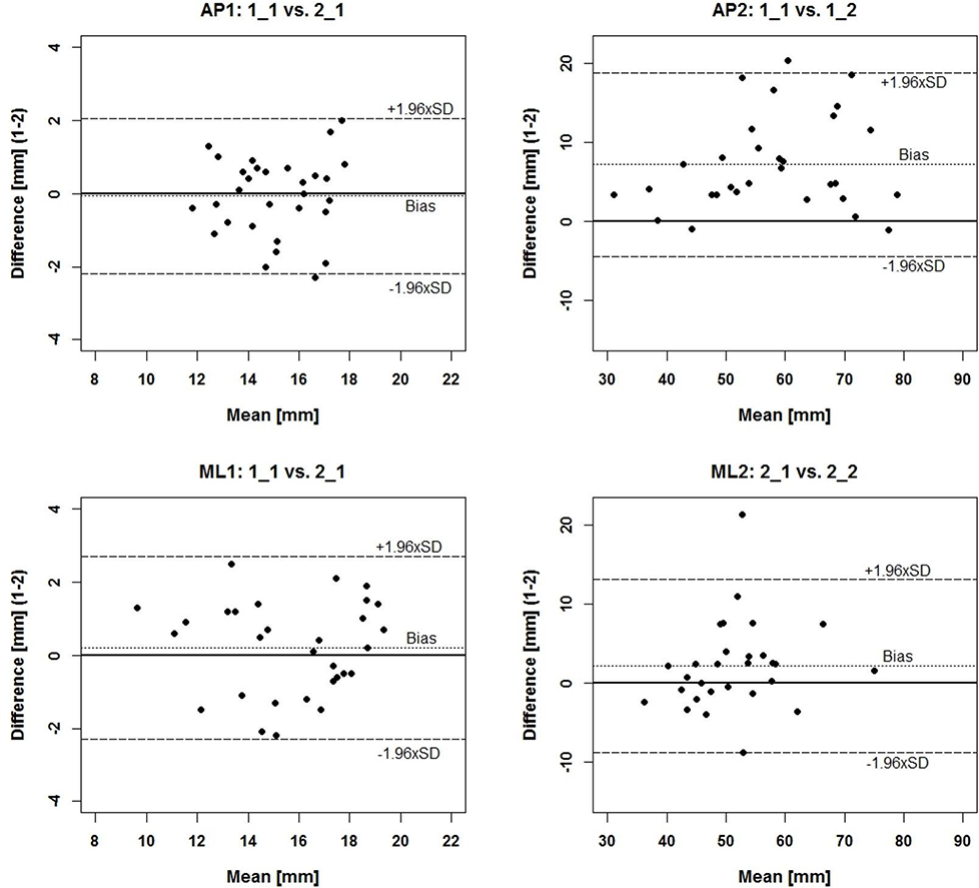
Bland-Altman plots for the AP (top) and ML (bottom) perturbation directions, showing examples for intervals 1 (left plots) and 2 (right plots).

Fig 3 shows exemplary Bland-Altman plots. Summarized data for all comparisons are provided in Table 2. Interval 1 generally demonstrated little bias and random error (Table 2) for both perturbation directions, slightly greater bias was observed for intra-day than for inter-day data.

**Table 2:** Overview of ICCs and parameters implemented in the analysis of Bland-Altman plots. Depicted are the grand mean (mean of both measurement sessions), bias, random error component and upper/lower limits of agreement (ULOA/LLOA, respectively) for inter- and intra-day comparisons of all intervals in the AP and ML direction.

**Int 1**	**1_1 vs. 1_2**		**2_1 vs. 2_2**		**1_1 vs. 2_1**		**1_2 vs. 2_2**	
[mm]	**AP**	**ML**	**AP**	**ML**	**AP**	**ML**	**AP**	**ML**
ICC	0.953	0.970	0.937	0.926	0.905	0.941	0.869	0.947
grand mean	14.9	15.5	14.9	15.3	15.1	15.7	14.8	15.1
bias	0.3	0.7	0.5	0.7	-0.1	0.2	0.1	0.2
random error	1.5	1.8	1.9	2.7	2.2	2.5	2.7	2.4
ULOA	1.8	2.5	2.3	3.4	2.0	2.7	2.7	2.6
LLOA	-1.3	-1.2	-1.4	-2.0	-2.2	-2.3	-2.6	-2.2
**Int 2**	**1_1 vs. 1_2**		**2_1 vs. 2_2**		**1_1 vs. 2_1**		**1_2 vs. 2_2**	
[mm]	**AP**	**ML**	**AP**	**ML**	**AP**	**ML**	**AP**	**ML**
ICC	0.942	0.888	0.934	0.880	0.751	0.713	0.844	0.853
grand mean	57.8	50.0	54.7	51.4	59.0	51.7	53.5	49.7
bias	7.2	2.1	4.0	2.1	4.9	-1.3	1.6	-1.4
random error	11.6	9.1	10.5	11.0	21.2	15.1	16.5	10.9
ULOA	18.8	11.2	14.5	13.1	26.1	13.7	18.2	9.5
LLOA	-4.5	-7.0	-6.5	-8.8	-16.4	-16.4	-14.9	-12.3

Bland-Altman parameters for interval 1 revealed that differences were located within the LOAs for 97% of all cases, for AP and ML. In two out of eight Bland-Altman plots, differences were not evenly distributed around the zero line for AP and ML (not graphically illustrated). Exemplary data for interval 1 are shown in Fig 3 (left graphs). For interval 2 (exemplary data in Fig 3, right graphs) in the ML direction, bias was low, although somewhat higher for intra-day than for inter-day comparisons (see Table 2). Differences were again mainly distributed within the LOAs, however, intra-day comparisons showed approx. 60% positive differences. For AP, three out of four plots presented large bias and more positive differences for intra-day comparisons, reflecting decreased COP excursions at the respective retest. Random error components were greater than in interval 1 when compared to the grand means (Table 2).

Table 2 summarizes ICC coefficients for intra- and inter-day comparisons and all intervals, ranging between 0.713 and 0.970.

## Discussion

This study aimed to analyze inter- and intra-day reliability of dynamic balance responses after unexpected perturbations using the Posturomed device. Various statistical approaches were used to assess reliability. In this regard, intra- and inter-day comparisons were made, because different intervention protocols are often conducted between two balance tests. Furthermore, intra-session trials between and within subjects were analyzed. As already shown, the Posturomed is also used as a training device [28], and therefore, consecutive balance tests might be prone to balance improvements at the retest. At the same time, the Posturomed is a standardized device to induce unexpected perturbations [12], hence also used to quantify dynamic balance ability [12]–[17].

The present study showed good relative and absolute reliability for both analyzed intervals and both perturbation directions. However, interval 2 exhibited slight learning effects when considering intra-day comparisons in the AP perturbation direction.

### Background on Analyzed Intervals

This section describes the physiological background regarding the time intervals analyzed in this study. As already mentioned, perturbations were implemented unexpectedly, eliciting compensatory motor reactions of muscles [6]. Stelmach et al. [29] examined postural reflexes for different perturbation conditions, similar to ones used in the present study. They found that the tibialis anterior muscle showed activity 100 ms after the onset of the perturbation. Other studies also implemented similar translational perturbations and found muscle latencies to be present at around 100 ms post perturbation [30], [31]. As a consequence of these muscular responses, it is obvious that active COP (Center of Pressure) displacements also occur. In this context, Müller and Redfern [31] examined similar anterior translational platform perturbations and showed that the onset of active COP displacements occurred at approx. 130 ms post perturbation onset. Consequently, active COP displacements in our study are present in interval 2, characterizing first compensatory reflex responses caused by the unexpected perturbation itself. Active balance responses are meaningful to assess dynamic balance, since they are important to control balance after sudden perturbations and hence prevent falls [32]. In contrast, during interval 1 (up to 70 ms post perturbation onset), muscular responses were rather a consequence of quasi-static balance demands of the previous pre-trigger time interval. However, it is possible that previous anticipatory activity still changed postural motor behavior in interval 1.

### Interval 1

The subjects’ ranges indicate that there were some variations of COP magnitudes between and within subjects. However, these magnitudes did not change within the 12 trials in each of the four measurement sessions (Fig 1). This indicates that there was no learning effect for AP or ML direction and was confirmed when considering inter- and intra-subject variations. In contrast, another study found slight and non-significant decreases of pathways within the course of five trials [22]. This study also used the Posturomed, but the platform pathway was analyzed for six seconds and the setup did not include unexpected perturbations.

Intra- and inter-day observations showed no significant bias for AP. In the ML direction, significant biases were found between 1_1 vs. 1_2 and 1_1 vs. 2_2. However, for both comparisons, differences accounted for only 0.7 mm (4.4%). Furthermore, since the root mean square error (RMSE) term calculated over these comparisons was larger than the mean differences, significant bias found here was not considered relevant regarding improving or deteriorating balance responses.

Considering means of each measurement session for each subject (Fig 2), no trend towards either increasing or decreasing excursions was evident. Additionally, intra- and inter-subject variations were very small (Fig 2). This means, following visual inspection, no learning effects seemed to be present.

ICC magnitudes found in interval 1 were considered to be high (>0.90, [33], [34]) and good (>0.70, [35]). High magnitudes of ICC coefficients show that the ranking of the subjects was similar for both intervals and all measurement sessions. This provides important information of how the measures are associated during several test-retest comparisons. The ICC model of this study (3, k) was only sensitive to random error [27], but the detected significant biases for ML were not relevant, hence ICC magnitudes indeed seem to reflect high relative reliability. Relative reliability describes how data or a score of the individual subject keeps its position with respect to the entire sample throughout repeated measurements [25]. However, relative reliability does not necessarily provide information on whether measures are close to each other within consecutive trials for individual subjects [25]. Therefore, absolute reliability was also assessed using Bland-Altman plots to indicate to what extent repeated measures change for individuals [25].

Little bias and an even distribution of differences around the zero line were observed in the Bland-Altman plots, pointing towards no learning effects. Bland-Altman parameters were interpreted as follows [36]: if a subject obtained an average COP Total excursion of 13.2 mm on the first test in AP, data could vary within the range of the LOAs (+1.8 and-1.3 mm), which is from 11.9 to 15.0 mm on a retest [36]. The amount of variation within 12 trials ranged from 10.8 to 17.1 mm for this subject. This demonstrates that variation within single trials is of greater magnitude than variation based on calculations using means and LOAs. Hence, the interpretation of the variation should not be based upon whether only a few differences fall outside the LOAs [33], but to evaluate the amount of variation during each of the 12 trials. Some of this variation seems to represent physiological balance patterns, e.g. anticipatory activity, which cannot be avoided. Other studies based on quasi-static balance tests found high intra-subject variations for consecutive days [37], [38] as well as within one day [38]. They suggest the high variability might be due to different balance strategies, and not necessarily due to a low reliability of the setup [38]. This presumption also agrees with Corriveau et al. [39], who estimated test-retest reliability in quasi-static double leg stance. It was assumed that measurement error is mainly linked to the biologic variability [39]. Similarly, Müller [40] found low oscillating frequency variations of the Posturomed, hence also suggesting that platform pathway depends on the subjects neuromuscular strategy. Note, however, that neuromuscular reactions due to the perturbation itself did occur in interval 2 (after approx. 100 ms [31]).

From all intra- and inter-day comparisons, there was only one exception in ML (1_1 vs. 1_2): slightly decreasing COP excursions were present indicating balance improvements at 1_2. The reason for this exception, however, is unknown. Considering the amount of intra- and inter-subject variability and the non-relevance of mean differences, differences should not be over-interpreted. Taking into account all analysis during interval 1, which mainly consisted of passive balance demands, data appear to be reliable. This means that the Posturomed indeed seems to be a standardized perturbation setup to induce unexpected translatoric perturbations, as also proposed by Kiss [12].

### Interval 2

No generally decreasing or increasing magnitudes were observed descriptively within all 12 trials per session, indicating neither improving nor deteriorating dynamic balance responses. Boeer et al. [22] examined balance ability using the Posturomed, however measuring total platform pathway with no perturbation during single leg stance. They performed retests after one to three weeks and found slight but not significant decreases within the course of five consecutive trials. In the present study, when comparing trials from 1_1 with 1_2, no trend was observable for AP or ML. However, especially for AP, decreased excursions at 1_2 (retest) were observed, indicating possible intra-day learning effects (Fig 1, subject 22: 1_1 vs. 1_2).

Mean COP Total excursions exhibited no significant bias for the ML direction, but all intra-day considerations and 1_1 vs. 2_2 for AP showed significant bias. Mean differences ranged from 3.7 to 8.6 mm, which corresponds to approx. 6.5 and 10.6% of the grand means, respectively. Similarly to interval 1, calculated RMSE values were larger than mean differences, meaning significant bias found here cannot be regarded as relevant. Therefore, with respect to mean COP excursions, no relevant balance improvements nor deteriorations were observed. This is in accordance with Corriveau et al. [39], who estimated test-retest reliability of center of pressure—center of mass variables. They also detected significant, but not relevant bias in the AP direction. However, they tested older subjects using both legs and measurements lasting for 120 seconds. With respect to the difference between older and younger subjects, Allum et al. [41] found a delayed (approx. 20 ms) onset of muscle responses for older subjects after unexpectedly induced roll and pitch perturbations. Since young subjects participated in the present study, our results might not be directly transferable to older populations. Further studies should investigate this aspect.

With regard to means of each measurement session for each subject, some presented higher COP excursions for AP at 1_1 and 2_1, when compared to the retests (1_2, 2_2, respectively). This means that those subjects improved their balance, especially within one day. This tendency was not detected for ML (Fig 2).

ICC coefficients of the present study (0.713 to 0.942) indicated good to high relative reliability [33]–[35]. Corriveau et al. [39] found moderate (0.72; 0.74) to excellent (0.89; 0.90) ICC coefficients when assessing quasi-static balance. Importantly, ICCs can be dependent on the variance or variability between single subjects [26], [27], [35], [42], [43] and, therefore, can be context-specific [44]. This means, high inter-subject variability most likely results in high ICC values [25], [27]. To better identify variability, coefficients of variation (COV) were also calculated. Some high ICC coefficients were found, although COVs had lower magnitudes (interval 1 AP, 1_1 vs. 1_2: ICC = 0.953, COV = 0.120) and vice versa (interval 2 AP, 1_1 vs. 2_1: ICC = 0.751, COV = 0.220). These considerations indicate that high ICC coefficients obtained in the present study did not necessarily occur due to high variability between subjects. Hence, good/high ICC magnitudes for interval 2 indeed seem to reflect good to high relative reliability. This indicates that, although during interval 2 there were biological variations due to motor responses, subjects maintained their position within the different test-retest configurations.

Bland-Altman plots for ML also show that the range occurring within 12 trials was larger than the range calculated with means (LOA), indicating high absolute reliability. The same can be claimed for AP, however, the presence of substantial bias became evident. Fig 3 shows that 93 and 80% of the subjects improved balance responses for both intra-day comparisons in AP, which is evident by the smaller COP excursions [45], [46] at both retests. For inter-day comparisons, this finding was not as pronounced. This might be explained in that the central nervous system elaborates a special motor memory program to improve reactive stability [32]. It might be supposable that the large time period between the tests (48 hours) led to a reduced ability of the subjects to recall the acute motor memory program from the pretest.

This means, for AP and especially for intra-day considerations, the present balance data seem to exhibit low absolute reliability, as also confirmed by relatively high random error components. A possible explanation for this is the difference between the limits of the base of support when comparing AP and ML perturbations: in AP, due to the anatomical foot structure, a greater COP path length enables the generation of greater torque compared to ML. Carpenter et al. [47] confirmed that perturbations in the ML direction are more challenging because they require a more complex muscle response program in comparison to perturbations in the AP direction. This consequently results in a greater demand on information processing in the central nervous system [41]. The less balance-challenging AP direction might induce greater potential to correct and improve balance after unexpected perturbations [40], hence allowing for biological variability. In contrast, Corriveau et al. [39] found lower reliability for the ML direction compared to the AP direction. However, our results may not be directly comparable to their study, since they measured older subjects (minimal age: 60 years) performing double limb stance. As already mentioned, absolute intra-day reliability was somewhat lower in the present study, especially in AP. This finding does not agree with Lin et al. [48], who found better absolute as well as relative reliability for intra-day compared to inter-day considerations. In contrast, their subjects performed quasi-static and upright double leg stance on four different days. A similar study examining balance ability and reliability (retests after 1 to 3 weeks) using the Posturomed found slightly smaller pathways at the retest, indicating possible, although minimal, learning effects [22]. It is also important to note that their study did not mention the relevance of the differences found.

Interval 2 shows high absolute reliability for inter-day comparisons, indicating that individual balance responses are similar for individual subjects during different test-retest comparisons. Lower absolute reliability was found for intra-day comparisons, especially in the AP direction, indicating possible learning effects.

## Conclusions

To summarize, when looking at each individual measurement session (12 trials) for all intervals, it is important to note that no increasing or decreasing excursions were observed. This indicates that there was no observable learning effect within the 12 trials performed. Therefore, future investigations using the Posturomed to assess dynamic balance responses should be able to use 12 trials without creating any learning effects. When considering mean COP excursions over those 12 trials for every subject and all intervals, significant differences between measurement sessions were detected in some cases, however, none were considered to be relevant. Furthermore, ICC coefficients were of high magnitude. Taking these considerations together, one would presume a generally good reliability for the intervals analyzed in this study. This is true for the majority of the present data, but there were some exceptions when analyzing inter- and intra-day reliability using Bland-Altman plots and inspecting individual trial data of the sessions. In this regard it was shown that intra-day reliability for interval 2 in the AP direction presented decreased excursions for the majority of subjects at the retests, corresponding to possible learning effects. For this reason, it is important to be aware of potential learning effects when performing examinations in the morning and afternoon of the same day, as conducted in the present study. These potential learning effects, however, did not occur between inter-day measurements within the intervals of this study. Additionally, it is important to note that our results should be acknowledged in the context of unexpectedly induced perturbations as performed in this study. Further studies should also investigate reliability aspects of muscular activity and when implementing longer test-retest periods. Moreover, it is also suggested to explore reliability aspects of dynamic balance responses of older subjects.

## Supporting Information

S1 File Minimal data set. (XLSX)
